# Efficacy of live attenuated, vector and immune complex infectious bursal disease virus (IBDV) vaccines in preventing field strain bursa colonization: A European multicentric study

**DOI:** 10.3389/fvets.2022.978901

**Published:** 2022-09-12

**Authors:** Gema Ramon, Matteo Legnardi, Mattia Cecchinato, Christophe Cazaban, Claudia Maria Tucciarone, Laura Fiorentini, Lorenzo Gambi, Tamas Mato, Giacomo Berto, Kostas Koutoulis, Giovanni Franzo

**Affiliations:** ^1^Ceva Santé Animale, Libourne, France; ^2^Department of Animal Medicine, Production and Health (MAPS), University of Padua, Padova, Italy; ^3^Istituto Zooprofilattico Sperimentale della Lombardia e dell'Emilia Romagna, Forlì, Italy; ^4^Istituto Zooprofilattico Sperimentale della Lombardia e dell'Emilia Romagna, Brescia, Italy; ^5^Scientific Support and Investigation Unit, Ceva-Phylaxia Co. Ltd., Ceva Animal Health, Budapest, Hungary; ^6^Ceva Salute Animale, Agrate Brianza, Italy; ^7^Department of Poultry Diseases, Faculty of Veterinary Science, University of Thessaly, Trikalon, Greece

**Keywords:** IBDV, vaccine, field studies, vector vaccines, immune complex (icx)

## Abstract

Infectious bursal disease virus (IBDV) is among the most relevant and widespread immunosuppressive agents, which can severely damage poultry farming by causing direct losses, predisposing the host to secondary diseases and reducing the efficacy of vaccination protocols against other infections. IBDV has thus been the object of intense control activities, largely based on routine vaccination. However, the need for protecting animals from the infection in the first period of the production cycle, when the bursa susceptibility is higher, clashes with the blanketing effect of maternally derived antibodies. To overcome this issue, other strategies have been developed besides live attenuated vaccines, including vector vaccines and immune complex (icx) ones. The present study aims to investigate, in field conditions, the efficacy of these approaches in preventing IBDV infection in laying chickens vaccinated with either live attenuated, vector or immune complex (icx) vaccines. For this purpose, a multicentric study involving 481 farms located in 11 European countries was organized and IBDV infection diagnosis and strain characterization was performed at 6 weeks of age using a molecular approach. Vaccine strains were commonly detected in flocks vaccinated with live or icx vaccines. However, a significantly higher number of field strains (characterized as very virulent IBDVs) was detected in flocks vaccinated with vector vaccines, suggesting their lower capability of preventing bursal colonization. Different from vector vaccines, live and icx ones have a marked bursal tropism. It can thus be speculated that vaccine virus replication in these sites could limit vvIBDV replication by direct competition or because of a more effective activation of innate immunity. Although such different behavior doesn't necessarily affect clinical protection, further studies should be performed to evaluate if vvIBDV replication could still be associated with subclinical losses and/or for viral circulation in a “vaccinated environment” could drive viral evolution and favor the emergence of vaccine-escape variants.

## Introduction

Immunosuppression is one of the most relevant threats to the poultry industry, which has increased along with the development of intensive farming. Among the several factors that can cause this pathological condition, viral infections play a major role. These infections can be extremely detrimental, not only due to direct losses, but especially because they increase the susceptibility to other pathogens and might cause the failure or reduction in the effectiveness of vaccination procedures. This can have severe consequences on animal health, welfare and productivity but also on human health since zoonotic infections can be favored (1, 2). Additionally, an increase in antimicrobial usage may be required (3).

Among the most relevant and widespread diseases causing immunosuppression is infectious bursal disease (IBD), also known as Gumboro disease. Its aetiological agent, named infectious bursal disease virus (IBDV), is a member of the *Birnaviridae* family, genus *Avibirnavirus* with a double-stranded RNA genome made of two segments. Segment A encodes the structural proteins VP2, VP3 and VP4 plus the regulatory protein VP5, while segment B encodes the viral polymerase VP1. VP2 is the main component of the viral capsid, contains the major immunogenic domains, and is the most relevant determinant of pathogenicity (4). Nonetheless, VP1 is also recognized to play a role in the latter (5).

Since the virus targets the immature B lymphocytes, IBDV infection causes the destruction of lymphoid organs, particularly the bursa of Fabricius, leading to immunosuppression (6). IBD is featured by not specific signs such as depression, dehydration and diarrhea, while macroscopic lesions are more pathognomonic and include hemorrhages in the thighs and pectoral muscles and bursal alterations, usually an initial enlargement with edema and mucosal petechiae which then evolves into atrophy (4). However, significant heterogeneity is observed between different IBDV strains in terms of genetic, pathogenic and antigenic features, which may affect the clinical presentation of the disease. The first strains to be characterized after the description of the disease, which occurred in Delaware in the 1960s, caused typical IBD signs and lesions with limited mortality, and were later known as classical strains (7). Variant strains differing in terms of antigenicity, mostly associated with subclinical infections and marked bursal atrophy, were described in the US in the 1980s, shortly followed by the emergence in Europe of the so-called very virulent strains, capable of causing outbreaks with significantly higher mortality (8). A plethora of other IBDV types have been subsequently described with increasing frequency, and are known to circulate either at global or more local level (9–14). This remarkable variability, which mostly originated from mutation and recombination mechanisms (15), is a crucial element to consider, as it may pose different sets of challenges toward the control of the disease.

IBDV control is hindered by its high infectiousness and resistance in the environment, which make biosecurity measures and good farming practices useful but usually not sufficient. Therefore, control is pursued mainly by extensive vaccination campaigns (2, 16). However, vaccination efficacy may be hampered by high levels of maternally-derived antibodies (MDA), which, despite being effective in protecting chickens during the first weeks, may inhibit the immune response to vaccination and cause poor protection in the following stages, especially when vaccination is performed with mild strains (17). Less attenuated live vaccines, like intermediate and especially intermediate plus and hot ones, are less affected by MDA but the increasingly high residual pathogenicity can lead to non-negligible damages to lymphoid tissues and associated decrease of the immune response against other vaccines or pathogens if administered too early (2). Therefore, a careful choice must be done, taking safety and efficacy into consideration.

The challenge in defining the best timing combined with the trade-off between MDA escape and virulence complicates the vaccine application and has determined a great variability of the implemented protocols, with multiple interventions at different ages, depending on the epidemiological scenario and/or to subjective decisions. Ideally, vaccination should be able to protect after a single dose, administered either “in ovo” or at hatching, regardless of MDA levels. Currently, two new-generation vaccines adequately satisfy such targets: vector vaccines, based on turkey herpesvirus carrying the IBDV VP2 protein, and immune complex vaccines (icx) (18, 19). It has been shown in different comparative studies that MDA interfere with humoral response in vaccination with intermediate live vaccines but do not have any impact on immunization with HVT recombinant vector vaccines (2, 20, 21). Icx consists of a mix of a vaccine virus and hyperimmune neutralizing antibodies in a concentration not able to actually neutralize the virus but delaying its replication and pathological effects, allowing the use of vaccine strains that would be otherwise too virulent in young animals. The coating of the virus prevents the neutralization of the vaccine virus from MDA and at the same time immunocomplexes are captured by follicular dendritic cells, which will progressively release it without antibody coating. The virus will be neutralized and won't start to replicate in presence of high MDA, while protection will start to rise parallelly with decreasing MDA (2).

Both vector and icx vaccines have been reported to be highly effective in protecting chickens against challenge, and appear particularly beneficial to the farming of commercial layers and slow-growth broilers, whose greater genetic susceptibility to IBD requires live attenuated vaccines to be administered multiple times by drinking water to ensure proper protection, often with variable results (22–24). Nevertheless, other aspects, including the capability of vaccines to prevent bursa colonization by other virulent strains, have been poorly investigated, especially in field conditions. The present multicentric study aims to evaluate the efficacy of different vaccination strategies, including live-attenuated vaccines, icx and vector vaccines in preventing bursal colonization.

## Materials and methods

### Sampling and metadata collection

Bursa samples were collected from 481 laying chicken farms located in 11 European countries (i.e., Austria, Czech Republic, France, Germany, Greece, Hungary, Italy, Netherlands, Poland, Portugal and Spain) at 6 weeks of age, in the period May 2018-September 2019. Among the sampled farms, 76 flocks were vaccinated with live attenuated vaccines, 149 with icx and 256 with vector vaccines. None of the flocks included in the study displayed clinical signs or lesions ascribable to IBDV infection. Since this was a field study and animals are routinely vaccinated in field conditions, including unvaccinated flocks was not considered in this study.

All procedures were performed in the framework of routine monitoring activity performed by CEVA. All involved veterinarians and technicians were employees of the company, who received specific training on the approach for samples and data collection. Particularly, a detailed protocol was developed and shared among the national branches of the company involved in the experimental procedure (SOP #VT04.18). Briefly, bursal sample had to be frozen −20°C after collection and delivered to the reference lab within 2 months. The following minimum information was registered for each sample: farm location, date, animal age and applied vaccination program.

Since the samples were collected within the context of routine diagnostic and monitoring activities and not for experimental purpose, no ethical approval was required. After collection, ten bursa samples were merged in pools, homogenized and stored at−80°C until processing.

### IBDV diagnosis and characterization

Samples collected in different countries were delivered to a single laboratory, owned by the company (Ceva Phylaxia, Budapest, Hungary) that applied the same diagnostic procedure to all the samples (25). The sample processing approach had been previously validated and routinely applied by the laboratory, which operated in fulfilling the good laboratory practices.

RNA was extracted from pooled samples homogenated using the High Pure Viral RNA Kit (Roche) according to manufacturer instructions and IBDV genome presence was assessed with the RT-PCR, as previously described Eterradossi et al. (26).

Successful amplification and specificity of the bands were verified through electrophoresis on SYBR safe stained 2% agarose gel and positive samples were Sanger sequenced using the same RT-PCR primers. Chromatogram quality was inspected using FinchTV (http://www.geospiza.com) and consensus sequences generated with ChromasPro (ChromasPro Version 2.0.0, Technelysium Pty Ltd, South Brisbane, Australia). Finally, strain characterization was performed according to the classification system proposed by Michel and Jackwood (27) by comparing the detected VP2 sequences to a set of reference strains (including the vaccine strains applied in the considered farms).

### Statistical analysis

All statistical analyses were performed in the R environment, benefitting of the suited libraries.

Since a huge number of different IBDV vaccination schemes are applied in Europe, the following aggregated categories were created to avoid sparse data:

- classical live-attenuated vaccines
- immune complex vaccines
- vector vaccines

Similarly, RT-PCR results and strain characterization were categorized in:

- Negative
- Vaccine strain (i.e. when the vaccine applied in the considered farm was sequenced at 6 week of age)
- Field strain

The association between vaccination strategy and the detected strain was assessed through the Chi-square test, setting the statistical significance level at p < 0.05. Particularly, the association between applied vaccine and presence or absence of (1) IBDV; (2) IBDV field strains (i.e., Negative and Vaccine strains were aggregated in a single category); and (3) Field or Vaccine strains (analysis performed on positive samples only).

## Results

### IBDV testing and characterization

At 6 weeks of age 248 farms tested IBDV positive, including 199 vaccines and 49 field strains (Table 1; Supplementary Table 1). All vaccine strains belonged to genogroup one, which groups classical IBDVs, while the entirety of field strains fell within genogroup three and were thus characterized as very virulent.

**Table 1:** Count of samples tested for each country classified according to the results of the diagnostic test and strain characterization.

**Country**	**Negative**	**Vaccine**	**Field**
Austria	8	0	0
Czech Republic	3	0	0
France	46	94	4
Germany	8	4	0
Greece	0	8	0
Hungary	4	6	0
Italy	55	50	30
Netherlands	55	12	11
Poland	0	7	4
Portugal	24	0	0
Spain	30	18	0
**Total**	**233**	**199**	**49**

The column totals are provided in bold.

**Figure 1:**
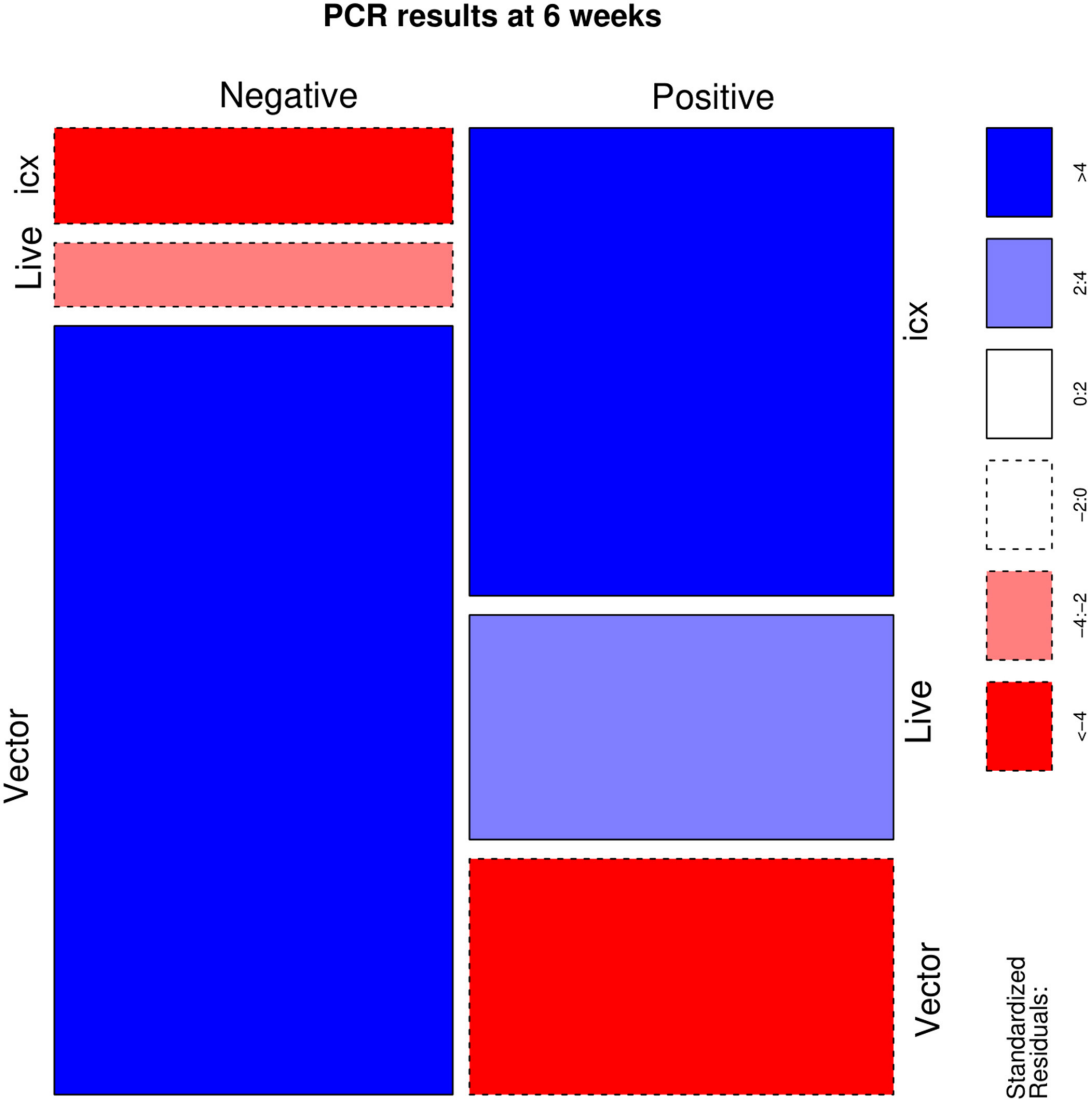
Mosaic plot depicting the relationship between the administered vaccine and the results of PCR assay. The area of each cell is proportional to the count size. Cells have been color-coded and lines dotted based on standardized residuals (a standardized residual >2 or < -2 is indicative of statistical significance).

**Figure 2:**
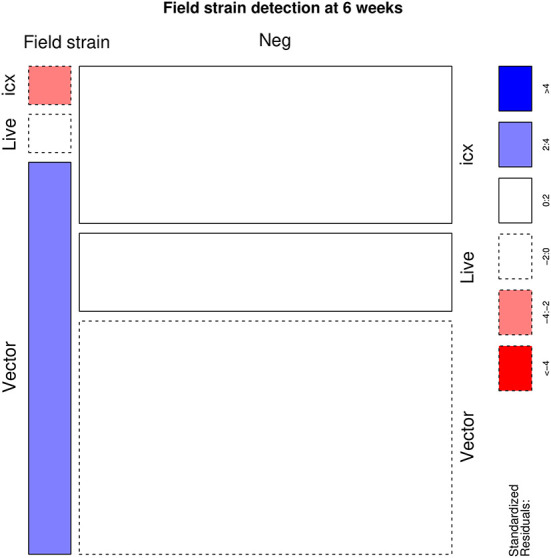
Mosaic plot depicting the relationship between the administered vaccine and the detection of field strains (i.e. samples negative to RT-PCR assay or characterizes as vaccine strains were merged in a single category). The area of each cell is proportional to the count size. Cells have been color-coded and lines dotted based on standardized residuals (a standardized residual >2 or < -2 is indicative of statistical significance).

A statistically significant association was identified (*p* < 0.001) between the applied vaccine and IBDV RT-PCR results. Particularly, flocks vaccinated with live and icx vaccines had a higher than expected number of positive samples. On the other hand, a lower frequency of positive samples was observed in flocks vaccinated with vector vaccines (Figure 1). However, the scenario reversed when IBDV negative and vaccine strains were aggregated in a single category, to evaluate the detection of field strains (Figure 2). An excess of field strains was observed in flocks vaccinated with vector vaccines, while these were underrepresented in those vaccinated with icx.

**Figure 3:**
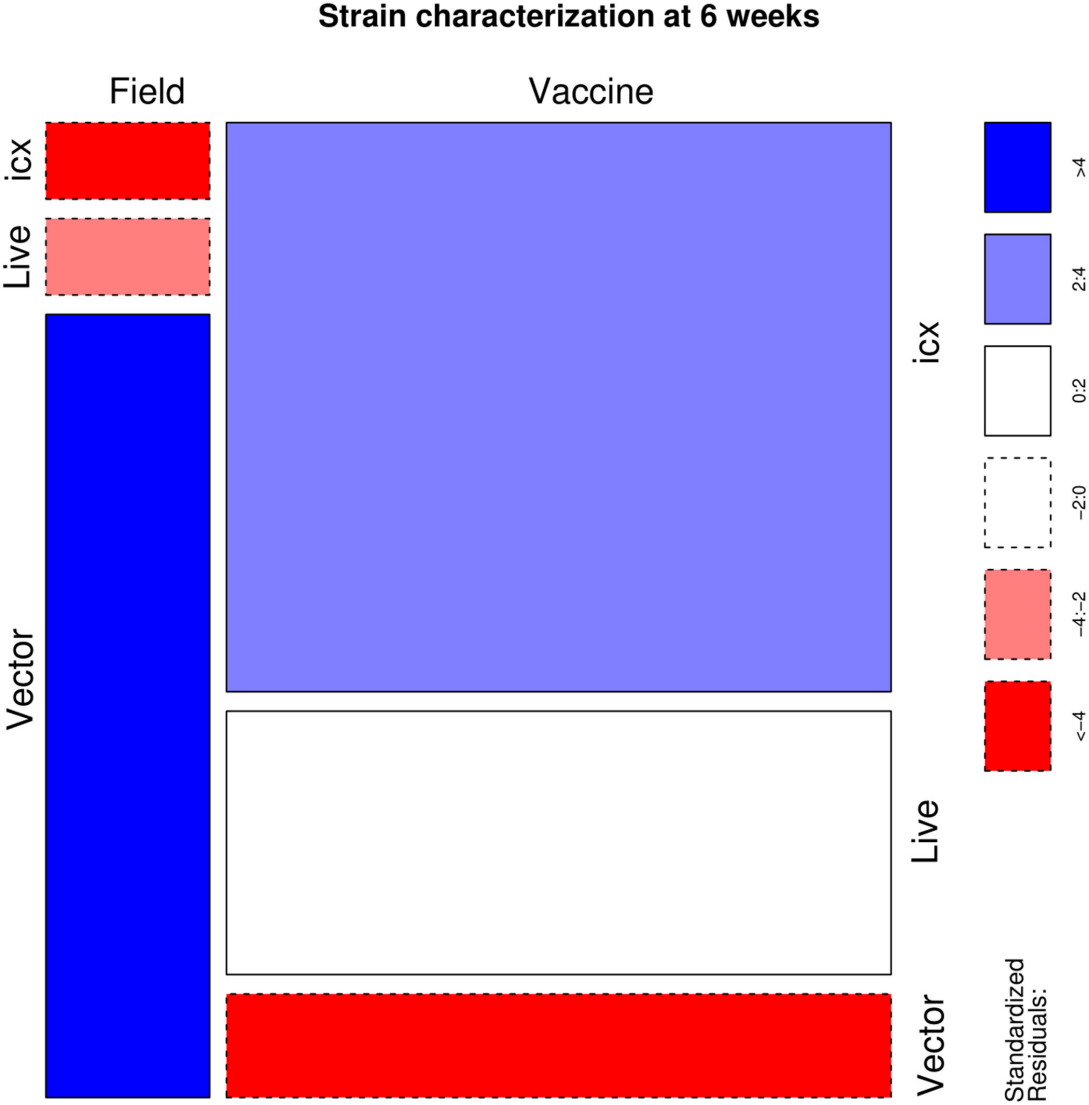
Mosaic plot depicting the relationship between the administered vaccine and the detection of field vs. vaccine strains. The area of each cell is proportional to the count size. Cells have been color-coded and lines dotted based on standardized residuals (a standardized residual >2 or < -2 is indicative of statistical significance).

Finally, when only positive samples were evaluated, a significant association (*p* < 0.001) was demonstrated between the applied vaccine and the detected IBDV strain. More in detail, an excess of field strains was detected in vector-vaccinated flocks, while a lower frequency was observed in those treated with live vaccines and especially in icx vaccinated ones. On the contrary, vaccine strains were underrepresented in flocks vaccinated with vector vaccines, while overrepresented in icx ones (Figure 3).

## Discussion

IBDV is still a major threat to the poultry industry in several countries. Besides direct losses, attributable especially to vvIBDV strains, variable decrease in immunocompetence, even when not clinically overt, can jeopardize the effectiveness of control measures against other diseases, reducing animal resistance and vaccine efficacy, thus leading to indirect losses, reduced animal welfare, decreased performance and higher use of treatments, like antimicrobials (2).

In the present study, 10% of the samples collected around Europe tested positive for IBDV field strains, belonging to the genogroup three. Although pathogenicity trials were not performed, and a reliable correlation between genetic similarities and virulence hasn't been established yet, considering the close genetic relationship of the obtained sequences with reference vvIBDV strains we suggest that they could also share the same phenotype and biological features. Therefore, IBDV circulation in Europe is still relevant, although the actual consequences may be difficult to estimate due to the insidious nature of the disease. This finding is of particular relevance since the flocks included in the monitoring activity were all vaccinated. Even though no outbreaks or major clinical problems have been reported, the non-negligible circulation of field strains (i.e., 19.75% of the positive samples), all classified within genogroup three, despite vaccination and in presence of vaccine-derived immunity should be considered worthy of further investigation for several reasons.

The presence of a highly infectious pressure can expose other unvaccinated/unproperly vaccinated flocks to overt disease development. Similarly, being vaccination coverage lower than 100%, a relevant proportion of the population can still be highly susceptible. A precise diagnosis would be challenging in this case and secondary infection impact goes unnoticed since only a limited percentage of the flock would be involved. Moreover, viral replication, although at a low level, can still potentially damage the host immune system, predisposing to secondary infection, especially when other co-factors are in place.

In fact, subclinical infections have frequently been associated with economic losses whose quantification is often challenging (28).

Viral circulation in an immune or partially immune population could allow vaccine-induced selective pressures to select those strains that are more able to deal with the new immune environment and potentially lead to the emergence of vaccine-escaping variants (29).

Therefore, the ability of vaccines to limit viral circulation, besides clinical manifestations, can be of remarkable relevance.

The present study demonstrates that although no vaccine was able to fully prevent field strain infections, they were significantly more frequent in flocks where vector vaccines were applied compared to those where live attenuated, particularly immune complex ones, were administered. The immunopathogenesis underlying this phenomenon was not within the scope of the present study and could be further investigated in other studies, However, different experimental studies reported higher protection and lower bursal lesions in chickens vaccinated with vector vaccines (30). Therefore, a lower viral circulation would be expected when such vaccines are applied in the long term, which is in clear contrast with the present study evidence. Unfortunately, the challenge virus titre was not monitored in Sedeik et al., study as well as in several similar experimental trials (30).

Different from vector vaccines, live and immune complex ones have a marked tropism for the bursae tissue. It can be speculated that vaccine virus replication in these sites could limit vvIBDV replication by direct competition or because of a more effective activation of innate immunity. Dedicated experimental studies would be necessary to confirm such a hypothesis and understand the underlying immunological processes. Despite the causes, the present study results are in agreement with other trials reporting the absence of vvIBDV challenge virus in immune-complex vaccinated groups (18). Regretfully, field/challenge strain detection and quantification are not common practices in studies evaluating the safety of IBDV vaccines. Therefore, reliable evidence of the mechanisms behind the different behavior of live and vector vaccines is lacking and need further investigations. Although obscure in the causes, the consequences of field strain circulation in flocks where vector-vaccines had been applied are considered investigation-worthy. Although current experimental data demonstrates no detrimental effect on individual animals, being vector vaccines able to provide typically higher protection compared to live attenuated vaccines, the implication at the epidemiological level and the effects on viral evolution over time must be carefully considered by researchers, field veterinarians and animal managers because of the potentially detrimental implication on the overall production system in the long term. In summary, the present study aimed to investigate the epidemiological behavior of different vaccination strategies including live attenuated, vector, or icx vaccines in the field, and the efficacy of these approaches in preventing IBDV infection in laying chickens. Although the mechanisms behind the efficacy of these vaccines were not considered in the study, a significantly higher number of very virulent IBDVs was detected in flocks vaccinated with vector vaccines, suggesting their lower capability of preventing bursal colonization.

While field strains were rarely detected in live and icx vaccinated flocks, vaccine detection at 6 weeks of age was on the other hand extremely common, particularly in the latter. The delayed viral replication due to the presence of specific antibodies in the icx formulation can be considered a likely explanation of the longer vaccine viral persistence and could contribute to limit the colonization of bursa tissues by field strains, as previously mentioned. Nevertheless, potential risk related to replication of live viruses, however attenuated, should deserve further evaluation.

At the present state, different vaccination strategies appear to have different advantages and disadvantages and imply different risks from an epidemiological/evolutive perspective. Therefore, systematic and specifically planned studies should be performed to evaluate this tradeoff and potentially define guidelines to drive the choice of the best vaccine according to the specific scenario.

## Data availability statement

The raw data supporting the conclusions of this article will be made available by the authors, without undue reservation.

## Ethics statement

Ethical review and approval was not required for the animal study because all procedures were performed in the framework of routine monitoring activity performed by CEVA. Since the samples were collected within the context of routine diagnostic and monitoring activities and not for experimental purpose, no ethical approval was therefore required. Written informed consent for participation was not obtained from the owners because the diagnostic activity was required by the animal owner and no additional procedure was performed.

## Author contributions

Conceptualization: GR and KK. Data curation: GF, LG, and LF. Laboratory IBDV diagnosis and sequencing: TM and Ceva Phylaxia laboratory personnel. Results organization and dataset preparation: GF, GB, LG, and LF. Statistical analysis: GF. Investigation: GR and MC. Methodology: ML. Project administration and resources: GR, KK, and CC. Supervision: GF, KK, and MC. Visualization: CT. Writing—original draft: GF. Writing—review and editing: ML, KK, CC, and MC. All authors contributed to the article and approved the submitted version.

## Funding

This study received funding from Ceva Santé Animal. The funder was not involved in the study design, analysis, interpretation of data, the writing of this article or the decision to submit it for publication.

## Conflict of interest

Authors GR and CC were employed by Ceva Santé Animal. This study received funding from Ceva Santé Animal. The funder provided the technicians and the software for data collection; however, it was not involved in data analysis and interpretation. The remaining authors declare that the research was conducted in the absence of any commercial or financial relationships that could be construed as a potential conflict of interest.

## Publisher's note

All claims expressed in this article are solely those of the authors and do not necessarily represent those of their affiliated organizations, or those of the publisher, the editors and the reviewers. Any product that may be evaluated in this article, or claim that may be made by its manufacturer, is not guaranteed or endorsed by the publisher.
