# Supplementary material for: Efficacy of live attenuated, vector and immune complex infectious bursal disease virus (IBDV) vaccines in preventing field strain bursa colonization: A European multicentric study
Source: Front Vet Sci. 2022 Sep 12;9:978901. doi: 10.3389/fvets.2022.978901 (PMC9510747; doi:10.3389/fvets.2022.978901)
Supplement: Supplementary file 1 [file Table_1.DOCX]

|  | **Immunocomplex** | | | | **Live** | | | | **Vector** | | | |  |
| --- | --- | --- | --- | --- | --- | --- | --- | --- | --- | --- | --- | --- | --- |
| **Country** | **Negative** | **Field** | **Vaccine** | **Total** | **Negative** | **Field** | **Vaccine** | **Total** | **Negative** | **Field** | **Vaccine** | **Total** | **Total** |
| Austria |  |  |  |  |  |  |  |  | 8 |  |  | 8 | 8 |
| Czech Republic |  |  |  |  |  |  |  |  | 3 |  |  | 3 | 3 |
| France | 24 | 4 | 94 | 122 |  |  |  |  | 22 |  |  | 22 | 144 |
| Germany |  |  | 4 | 4 |  |  |  |  | 8 |  |  | 8 | 12 |
| Greece |  |  | 8 | 8 |  |  |  |  |  |  |  |  | 8 |
| Hungary |  |  |  |  |  |  | 6 | 6 | 4 |  |  | 4 | 10 |
| Italy |  |  |  |  | 15 |  | 40 | 55 | 40 | 30 | 10 | 80 | 135 |
| Netherlands |  |  | 12 | 12 | 1 |  |  | 1 | 54 | 11 |  | 65 | 78 |
| Poland |  |  |  |  |  | 4 | 7 | 11 |  |  |  |  | 11 |
| Portugal |  |  |  |  |  |  |  |  | 24 |  |  | 24 | 24 |
| Spain |  |  | 3 | 3 |  |  | 3 | 3 | 30 |  | 12 | 42 | 48 |
| **Total** | **24** | **4** | **121** | **149** | **16** | **4** | **56** | **76** | **193** | **41** | **22** | **256** | **481** |

Supplementary table 1. Count of samples tested for each country classified according to the administered vaccine and results of the diagnostic test and strain characterization.
